# Polyphenolic Characterization, Antioxidant, and Cytotoxic Activities of *Mangifera indica* Cultivars from Costa Rica

**DOI:** 10.3390/foods8090384

**Published:** 2019-09-02

**Authors:** Mirtha Navarro, Elizabeth Arnaez, Ileana Moreira, Silvia Quesada, Gabriela Azofeifa, Krissia Wilhelm, Felipe Vargas, Pei Chen

**Affiliations:** 1 Department of Chemistry, University of Costa Rica (UCR), Rodrigo Facio Campus, San Pedro Montes Oca, San Jose 2060, Costa Rica (K.W.) (F.V.); 2 Department of Biology, Technological University of Costa Rica (TEC), Cartago 7050, Costa Rica; 3 Department of Biochemistry, School of Medicine, University of Costa Rica (UCR), Rodrigo Facio Campus, San Pedro Montes Oca, San Jose 2060, Costa Rica (I.M.) (S.Q.) (G.A.); 4 Methods and Application of Food Composition Laboratory, Beltsville Human Nutrition Research Center, Agricultural Research Service, U.S. Department of Agriculture, Beltsville, MD 20705, USA

**Keywords:** *Mangifera indica*, *mango*, UPLC, ESI-MS, polyphenols, xanthonoids, gallotannins, hydroxybenzophenones, mass spectrometry, antioxidant, antitumoral

## Abstract

The phenolic profile of skin and flesh from *Manifera indica* main commercial cultivars (Keitt and Tommy Atkins) in Costa Rica was studied using ultra performance liquid chromatography coupled with high resolution mass spectrometry (UPLC-ESI-MS) on enriched phenolic extracts. A total of 71 different compounds were identified, including 32 gallates and gallotannins (of different polymerization degree, from galloyl hexose monomer up to decagalloyl hexoses and undecagalloyl hexoses); seven hydroxybenzophenone (maclurin and iriflophenone) derivatives, six xanthonoids (including isomangiferin and mangiferin derivatives); 11 phenolic acids (hydroxybenzoic and hydroxycinnamic acid derivatives); and eight flavonoids (rhamnetin and quercetin derivatives). The findings for T. Atkins skin constitute the first report of such a high number and diversity of compounds. Also, it is the first time that the presence of gallotannin decamers and undecamers are reported in the skin and flesh of Keitt cultivar and in T. Atkins skins. In addition, total phenolic content (TPC) was measured with high values especially for fruits’ skins, with a TPC of 698.65 and 644.17 mg gallic acid equivalents/g extract, respectively, for Keitt and T. Atkins cultivars. Antioxidant potential using 2,2-diphenyl-1-picrylhidrazyl (DPPH) and oxygen radical absorbance capacity (ORAC) methods were evaluated, with T. Atkins skin showing the best values for both DPPH (IC_50_ = 9.97 µg/mL) and ORAC (11.02 mmol TE/g extract). A significant negative correlation was found for samples between TPC and DPPH antioxidant values (*r* = −0.960, *p* < 0.05), as well as a significant positive correlation between TPC and ORAC (*r* = 0.910, *p* < 0.05) and between DPPH and ORAC antioxidant methods (*r* = 0.989, *p* < 0.05). Also, cytotoxicity was evaluated in gastric adenocarcinoma (AGS), hepatocarcinoma (HepG2), and colon adenocarcinoma (SW620), with T. Atkins skin showing the best results (IC_50_ = 138–175 µg/mL). Finally, for AGS and SW 620 cell lines particularly, a high significant negative correlation was found between cytotoxic activity and gallotannins (*r* = −0.977 and *r* = −0.940, respectively) while for the HepG2 cell line, the highest significant negative correlation was found with xanthonoids compounds (*r* = −0.921).

## 1. Introduction

Several studies have linked vegetable consumption, especially fruits, with a reduced risk for cardiovascular disease and cancer, thus the importance of metabolites’ characterization. Mango (*Mangifera indica* L.) is a commercial fruit cultivated worldwide that holds the fifth position in total production amongst the main fruit crops, with 5.4 million hectares in approximately 100 countries, especially in areas with subtropical and tropical climates [1]. Out of the large number of cultivars reported, Keitt and Tommy Atkins are the most important commercialized mango cultivars in Costa Rica. 

The bioactive effects reported for *M. indica* include antioxidant activity, anti-inflammatory, antipyretic, antibacterial, antiviral, antimicrobial, and anticancer, as well as hepatoprotective and gastroprotective properties, in addition to immunomodulatory and lipid-lowering drug effects [2,3,4,5]. Particularly, it has been reported that mango exhibits antiproliferative activity in MDA-MB-231 adenocarcinoma breast cell lines, HepG2 liver, and HL-60 leukemia cancer cells [6], as well as antitumoral effects on MCF-7 breast carcinoma cells [7], Molt-4 leukemia, A-549 lung, LnCap prostate, and SW-480 colon cancer cells [8]. These studies report different results depending on mango cultivar and on cancer cell lines; however, the effects have mainly been attributed to the fruits’ polyphenolic contents.

In fact, several studies have reported polyphenolics benefits on health based on findings from the above bioactivities [9] and have established their role in reducing the risk of degenerative and chronic diseases, therefore contributing to long-term health protection [10]. For instance, their contents in fruits have been associated to a lower risk for cardiovascular diseases and cancer, hence the increase in interest in fruit consumption and the importance of scientific research for polyphenols’ characterization and their associated valuable effects on health.

Previous studies of *M. indica* polyphenols have focused, for instance, on properties of xanthonoid compounds, mainly mangiferin isomers [11,12] and gallotannins [13], which have been studied for their anticarcinogenic effects [6,14]. Other studies have involved gallotannins and hydroxybenzophenones [15], xanthonoids and flavonoids [16,17], or phenolic acids and gallotannins [18]. Few reports have studied all five types of compounds [19,20] and their biological activities [21]. 

Polyphenols’ antioxidant properties have been linked, among others, with their anti-inflammatory and anticancer activities, which have been reported to increase with gallotannins’ degree of polymerization of specific structures, which has been found to enhance such properties [22], thus further knowledge on phenolic structures’ characterization in mango fruits would contribute to a better understanding of their implications in the fruits’ quality as a source of dietary compounds with potential biological properties.

Therefore, the objective of the present work was to obtain enriched polyphenolic extracts of fruits from *M. indica* commercial cultivars in Costa Rica and to characterize them through ultra performance liquid chromatography coupled with high resolution mass spectrometry (UPLC-DAD-ESI-MS), with an emphasis on the five types of compounds previously reported. An evaluation of the total polyphenolic contents and antioxidant activity using 2,2-diphenyl-1-picrylhidrazyl (DPPH) and oxygen radical absorbance capacity (ORAC) methods, as well as the cytotoxic activity (MTT) on the adenocarcinoma AGS gastric cell line, adenocarcinoma HepG2 hepatic cell line, and adenocarcinoma SW620 colon cell line was also carried out in the different extracts.

## 2. Materials and Methods

### 2.1. Materials, Reagents and Solvents

*Mangifera indica* fruits were acquired in the ripe state from a local producer from Marichal Orotina Orotina (Keitt cultivar) and Fabio Baudrit Station (Alajuela). Cultivars were confirmed with the support of the Costa Rican National Herbarium and vouchers are deposited there. Reagents, such as fluorescein, 2,2-azobis(2-amidinopropane) dihydrochloride (AAPH), 2,2-diphenyl-1-picrylhidrazyl (DPPH), Trolox, gallic acid, Amberlite XAD-7 resin, fetal bovine serum, glutamine, penicillin, streptomycin, amphotericin B, and trypsin–ethylenediaminetetraacetic acid (EDTA), were provided by Sigma-Aldrich (St. Louis, MO, USA). Human gastric adenocarcinoma cell line AGS, human colorectal adenocarcinoma SW 620, and human hepatocellular carcinoma Hep-G2 were obtained from American Type Culture Collection (ATCC, Rockville, MD, USA). In order to evaluate the specificity of the cytotoxic activity towards these cancer cells with respect to normal cells, a selectivity index (SI) was determined by also measuring the cytotoxicity on normal non-cancer cells, according to previous publications [23,24,25]. Different cell lines are used in the literature, such as normal mouse subcutaneous fibroblast L929 in studies evaluating cytotoxicity on HeLa and SiHa cervical cancer cells [26]; normal human dermal fibroblast TelCOFSO2MA used in comparative cytotoxicity studies with Caco-2 colon and OE19 esophageal adenocarcinoma cell lines [27] and normal monkey epithelial kidney Vero cells used as non-tumoral control cells in studies evaluating cytotoxicity towards MCF-7 breast and HeLa cervix cancer cells [24]; Caco-2 colon and A549 lung cancer cells [23]; AGS gastric and SW620 adenocarcinoma cells [28]; and malignant HepG2 hepatoma cells [25]. These Vero cell lines were selected for this study due to previous reports and accessibility (American Type Culture Collection, Rockville, MD, USA). Finally, solvents, such as acetone, chloroform, and methanol, were purchased from Baker (Center Valley, PA, USA), while DMSO was acquired from Sigma-Aldrich (St. Louis, MO, USA).

### 2.2. Phenolic Extracts from Mangifera. Indica Fruits

*M. indica* fruits were rinsed in water, peeled, and both the skin and flesh material were freeze-dried in a Free Zone at −105 °C, 4.5 L, Cascade Benchtop Freeze Dry System (Labconco, Kansas, MO, USA). The freeze-dried material was preserved at −20 °C until extraction. Freeze-dried samples were extracted in a Dionex™ ASE™ 150 Accelerated Solvent Extractor (Thermo Scientific™, Walthman, MA, USA) using methanol:water (70:30) as solvent for 7.5 g of sample in a 34 mL cell, at 40 °C. Next, the extract was evaporated under vacuum to eliminate the methanol and the aqueous phase was washed with ethyl acetate and chloroform to remove less-polar compounds. Afterwards, the aqueous extract was evaporated under vacuum to eliminate organic solvent residues and was eluted (2 mL/min) in an Amberlite XAD7 column (150 mm × 20 mm), starting with 300 mL of water to remove sugars, and then with 200 mL each of methanol:water (80:20) and pure methanol to obtain the polyphenols. Finally, the enriched extract was obtained after evaporation to dryness using a Buchi™ 215 (Flawil, Switzerland) rotavapor.

### 2.3. Total Phenolic Content

The polyphenolic content was determined by modification of the Folin–Ciocalteu (FC) method [29], which is based on the oxidation of the hydroxyl groups of phenols by the mixture of phosphotungstic and phosphomolybdic acids. Briefly, each polyphenolic enriched extract was dissolved in MeOH (0.1% HCl) to obtain a 500 ppm solution and 2 mL were combined with 0.5 mL of FC reagent. Afterwards 10 mL of Na_2_CO_3_ (7.5%) were added and the volume was completed to 25 mL with water. Blanks were prepared in a similar way but using 0.5 mL of MeOH (0.1% HCl) instead of the sample. The mixture was left standing in the dark for 1 h and then absorbance was measured at 750 nm. Values obtained were extrapolated in a gallic acid calibration curve. Total phenolic content was expressed as mg gallic acid equivalents (GAE)/g sample. Analyses were performed in triplicate.

### 2.4. UPLC-DAD-ESI-TQ-MS Analysis

The UPLC-MS system used to analyze the composition of *M. indica* fruit extracts consisted of an LTQ Orbitrap XL mass spectrometer with an Accela 1250 binary Pump, PAL HTC Accela TMO autosampler, PDA detector (Thermo Fisher Scientific, San Jose, CA, USA), and G1316A column compartment (Agilent, Palo Alto, CA, USA). Separation was carried out by a modification of a method previously described [30]. Briefly, a Hypersil Gold AQ RP-C18 UHPLC column (200 mm × 2.1 mm i.d., 1.9 µm, Thermo Fisher Scientific) with an UltraShield pre-column filter (Analytical Scientific Instruments, Richmond, CA, USA) were used at a flow rate of 0.3 mL/min. Mobile phases A and B consist of a combination of 0.1% formic acid in water, *v*/*v* and 0.1% formic acid in acetonitrile, *v*/*v*, respectively. The linear gradient is from 4% to 20% B (*v*/*v*) at 20 min, to 35% B at 30 min and to 100% B at 31 min, and held at 100% B to 35 min. The UV/Vis spectra were acquired from 200 to 700 nm. The mass spectrometer was calibrated using Pierce™ LTQ ESI Negative Ion Calibration Solution, and the conditions for the negative electrospray ionization mode used were set as follows: Sheath gas, 70 (arbitrary units); aux and sweep gas, 15 (arbitrary units); spray voltage, 4.8 kV; capillary temperature, 300 °C; capillary voltage, 15 V; and tube lens, 70 V. The mass range was from 100 to 2000 amu with a resolution of 30,000, FTMS AGC target at 2e5, FT-MS/MS AGC target at 1 × 10^5^, isolation width of 1.5 *amu*, and max ion injection time of 500 ms. A clean chromatographic separation was obtained, and the most intense ion was selected for the data-dependent scan to offer MS^2^ to MS^5^ product ions, respectively, with a normalization collision energy at 35%.

### 2.5. DPPH Radical-Scavenging Activity

DPPH evaluation was performed by a modification of the original method [31], based on antioxidant determinations using a stable free radical. Briefly, a solution of 2,2-diphenyl-1-picrylhidrazyl (DPPH) (0.25 mM) was prepared using methanol as the solvent. Next, 0.5 mL of this solution were mixed with 1 mL of each polyphenolic-enriched extract at different concentrations ranging between 4 and 40 ppm, and incubated at 25 °C in the dark for 30 min. The mixture absorbance was measured at 517 nm. Blanks were prepared for each concentration. The percentage of the radical-scavenging activity of the sample was plotted against its concentration to calculate IC_50_ (µg/mL). The samples were analyzed in three independent assays. Results were expressed as IC_50_ (µg/mL), which is the amount of sample required to reach 50% radical-scavenging activity.

### 2.6. ORAC Antioxidant Activity

The ORAC (oxygen radical absorbance capacity) antioxidant activity was determined by modification of a method using fluorescein as a fluorescence probe [32]. Briefly, the reaction was performed in 75 mM phosphate buffer (pH 7.4) at 37 °C. The final assay mixture consisted of AAPH (12 mM), fluorescein (70 nM), and either Trolox (1–8 µM) or the extract at different concentrations. Fluorescence was recorded every minute for 98 min in black 96-well untreated microplates (Nunc, Denmark), using a Polarstar Galaxy plate reader (BMG Labtechnologies GmbH, Offenburg, Germany) with 485-P excitation and 520-P emission filters. Fluostar Galaxy software version 4.11−0 (BMG Labtechnologies GmbH, Offenburg, Germany) was used to measure fluorescence. Fluorescein was diluted from a stock solution (1.17 mM) in 75 mM phosphate buffer (pH 7.4), while AAPH and Trolox solutions were freshly prepared. All reaction mixtures were prepared in duplicate and three independent runs were completed for each extract. Fluorescence measurements were normalized to the curve of the blank (no antioxidant). From the normalized curves, the area under the fluorescence decay curve (AUC) was calculated as:

(1) $$AUC=1+\sum_{i=1}^{i=98}\int_{i}/\int_{0},$$

where *∫*_0_ is the initial fluorescence reading at 0 min and *∫_i_* is the fluorescence reading at time *i*. The net AUC corresponding to a sample was calculated as follows:

Net AUC = AUC_antioxidant_ − AUC_blank_. (2)

The regression equation between the net AUC and the antioxidant concentration was calculated. The ORAC value was estimated by dividing the slope of the latter equation by the slope of the Trolox line obtained for the same assay. Final ORAC values are expressed as mmol of Trolox equivalents (TE)/g of phenolic extract.

### 2.7. Evaluation of Cytotoxicity of Extracts

#### 2.7.1. Cell Culture

The human gastric adenocarcinoma cell line AGS, human colorectal adenocarcinoma SW 620, human hepatocellular carcinoma Hep-G2, and monkey normal epithelial kidney cells Vero were grown in minimum essential Eagle’s medium (MEM) containing 10% fetal bovine serum (FBS) in the presence of 2 mmol/L glutamine, 100 IU mL^−1^ penicillin, 100 μg/mL streptomycin, and 0.25 μg/mL amphotericin B. The cells were grown in a humidified atmosphere containing 5% CO_2_ at 37 °C and sub-cultured by detaching with trypsin–EDTA solution at about 70% to 80% confluence. For the experiments, 100 μL of a cell suspension of 1.5 × 10^5^ cells/mL were seeded overnight into 96-well plates. The cells were further exposed for 48 h to various concentrations of extracts (50 μL), dissolved in DMSO, and diluted with cell culture medium to final concentrations between 15 and 500 μg/mL. The DMSO concentrations used in the experiments were below 0.1% (*v*/*v*) and control cultures were prepared with the addition of DMSO (vehicle control).

#### 2.7.2. Assessment of Cytotoxicity by MTT Assay

After incubation for 48 h, MTT assays were performed to evaluate cytotoxicity. Briefly, the medium was eliminated, cells were washed twice with 100 µL of PBS, and incubated with 100 µL MTT solution (3-(4,5-dimethylthiazolyl-2)-2,5-diphenyltetrazolium bromide, 5 mg/mL in cell culture medium) for 2 h at 37 °C. The formazan crystals formed were dissolved in 100 µL of ethanol 95% and the absorbance was read at 570 nm in a microplate reader. Dose–response curves were established for each extract and the concentration that is enough to reduce the cell viability by 50% (IC_50_) was calculated.

In order to evaluate if the cytotoxicity activity was specific against the cancer cells, a selectivity index (SI) was determined. This index is defined as the ratio of IC_50_ values of normal epithelial kidney cells (Vero) to cancer cells (AGS, HepG2, or SW620). 

### 2.8. Statistical Analysis

In order to evaluate if the total phenolic contents (TPC) contribute to the antioxidant activity evaluated with the DPPH and ORAC methodologies, a correlation analysis was carried out between the TPC values and the DPPH and ORAC results. Also, one-way analysis of variance (ANOVA) followed by Tukey’s post hoc test was applied to the TPC, DPPH, and ORAC values, and differences were considered significant at *p* < 0.05.

## 3. Results and Discussion

### 3.1. Phenolic Yield and Total Phenolic Contents

The extraction process described in the Materials and Methods section allowed the phenolic-enriched extracts to be obtained, which are expressed as g of phenolic enriched extract/100 g of dry material and are summarized in Table 1. Keitt cultivar skin presented the highest yield (2.77 g extract/100 g dry material) whereas Tommy Atkins flesh showed the lowest value (0.57 g extract/100 g dry material). In both cultivars, skin extract yields were higher than flesh extracts.

The total phenolic contents (TPC) summarized in Table 1 show results ranging between 162.7 and 698.7 gallic acid equivalents (GAE)/g dry extract. The one-way analysis of variance (ANOVA), with a Tukey post hoc as the statistical test, showed a significant difference (*p* < 0.05) between results for the skin and flesh of *M. indica* samples, with a much higher average for skins corresponding to 671.4 GAE/g dry extract compared to a three times lower average value of 226.9 GAE/g dry extract for flesh. Total phenolic contents (TPC) previous reports for the flesh of T. Atkins cultivars from Mexico and Spain range between 15.3 and 21.77 mg GAE/100 g FW [8,33], whereas our finding of 16.3 mg GAE/100 g FW (value calculated using TPC and lyophilization yields from Table 1) fits within that range. Meanwhile, values reported for the skins of T. Atkins (43.17 mg GAE/100 g FW) and Pica (72.01 mg GAE/100 g FW) cultivars from Chile [34] are lower than our result of 380.9 mg GAE/100 g FW (value calculated using TPC and lyophilization yields from Table 1). In respect to Keitt flesh, results from the literature show variability, reporting values ranging between 17.99 and 59.43 mg GAE/100 g FW for Keitt and other cultivars from Italy and China [21,33] whereas our result of 33.9 mg GAE/100 g FW (value calculated using TPC and lyophilization yields from Table 1) fits in that range. Finally, regarding Keitt skin, previous results for this and other cultivars from China range between 368.52 and 641.9 mg GAE/100 g FW [21]. Our finding of 402.5 mg GAE/100 g FW (value calculated using TPC and lyophilization yields from Table 1) fit within that range and are higher than values found for the Irwin cultivar (26.9 mg GAE/g extract) from Korea [35]. TPC content variations are linked to polyphenols present in extracts [36,37] and the influence of these metabolites in extracts’ biological properties, such as the antioxidant capacity [38,39] and cytotoxic activity [14,22], as discussed in the following sections.

### 3.2. Profile by UPLC-DAD-ESI-TQ-MS Analysis

**Figure 1:**
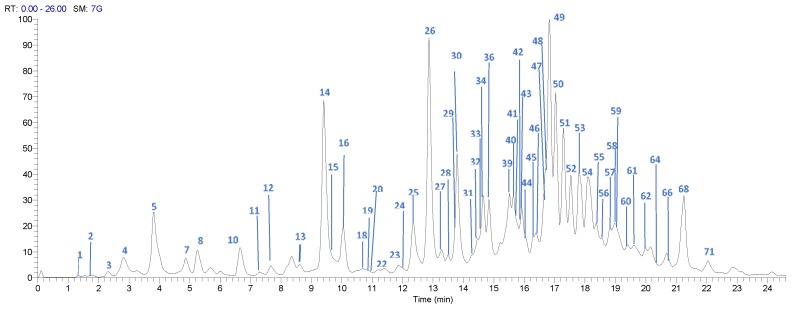
High Performance Liquid Chromatography (HPLC) chromatogram of *Mangifera indica* Tommy Atkins cultivar skin extract, in a Hypersil Gold AQ RP-C18 column (200 mm × 2.1 mm × 1.9 µm) using an LTQ Orbitrap XL Mass spectrometer (Thermo Scientific™, Walthman, MA, USA) in a mass range from 100 to 2000 amu.

**Figure 2:**
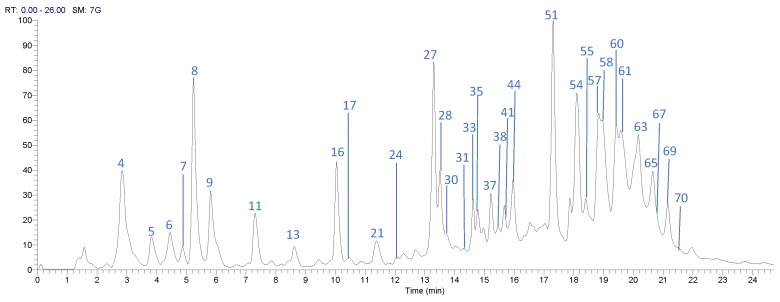
High Performance Liquid Chromatography (HPLC) chromatogram of *Mangifera indica* Keitt flesh extract in a Hypersil Gold AQ RP-C18 column (200 mm × 2.1 mm × 1.9 µm) using an LTQ Orbitrap XL mass spectrometer (Thermo Scientific™, Walthman, MA, USA) in a mass range from 100 to 2000 amu.

The UPLC-DAD-ESI-MS/MS analysis described in the Materials and Methods section allowed identification of 71 compounds, including 32 gallates and gallotannins, six xanthonoids, eight hydroxybenzophenones, eight flavonoids, and 11 phenolic acids and derivatives, in Costa Rican Keiit and T. Atkins commercial cultivars. Figure 1 and Figure 2 show the chromatograms of the 71 different compounds and Table 2 summarizes the results of the identification analysis.

#### 3.2.1. Benzoic and Hydroxycinnamic Acids 

**Figure 3:**
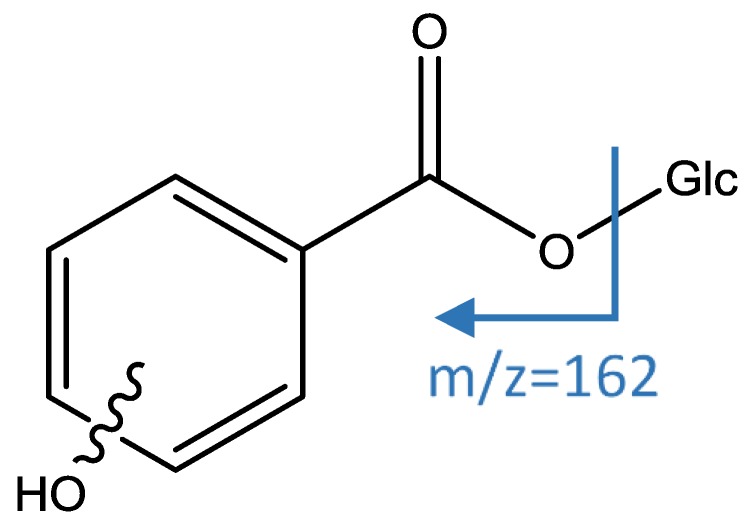
Structure and fragments of acid derivatives (6) and (9).

Peaks 6 and 9 had an [M−H]^−^ ion at *m*/*z* 299.0780 (C_13_H_15_O_8_) and a main MS2 fragment at *m*/*z* 137 [M−H−162]^−^ corresponding to a loss of hexose (Glc). Thus, those peaks were identified as isomers of hydroxybenzoic acid hexoside [40], as shown in Figure 3.

**Figure 4:**
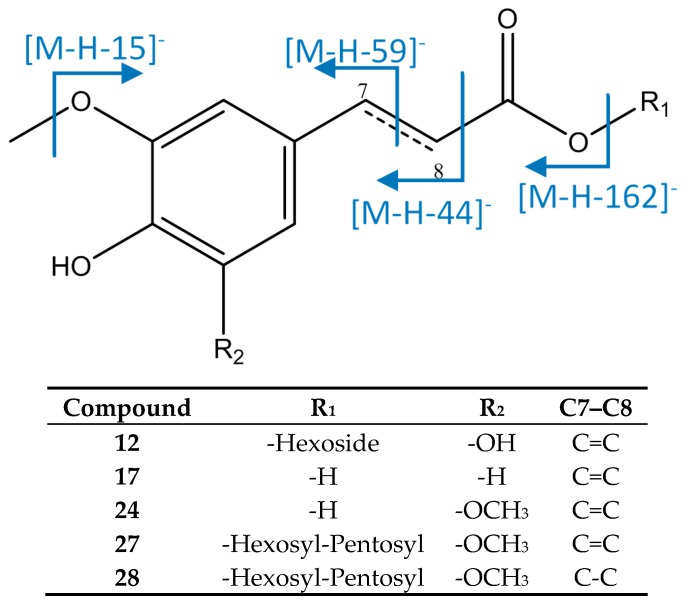
Structure and fragments of acid derivatives (12), (17), (24), (27), and (28).

As represented in Figure 4, peak 12 was identified as 5-hydroxyferuloyl hexoside, with [M−H]^−^ = 371.0993 (C_16_H_19_O_10_), with fragments at *m*/*z* 233 due to the fragmentation of the aromatic moiety, and at *m*/*z* 209 [M−H−162]^−^ due to loss of an hexoside. [41] Peak 17 had an [M−H]^−^ ion at 193.0515 (C_10_H_9_O_4_) that agreed with ferulic acid, with fragments at *m*/*z* 178, 149, and 134, due to a loss of methyl groups [M−H−15]^−^, carbon dioxide from the carboxylic acid [M−H−44]^−^, and cleavage through the double bond [M−H−59]^−^ [42]. Peak 24 was assigned to sinapic acid due to its [M−H]^−^ ion at 223.0618 (C_11_H_11_O_5_), which had main fragments at *m*/*z* 208 [M−H−15]^−^, 179 [M−H−44]^−^, and 164 [M−H−59]^−^. Peaks 27 ([M−H]^−^ = 517.2304, C_24_H_37_O_12_) and 28 ([M−H]^−^ = 519.2462, C_24_H_39_O_12_) were identified respectively as sinapic acid *O*-pentosyl-hexoside and dihydrosinapic acid *O*-pentosyl-hexoside, which showed an MS2 fragment at [M−H−132]^−^ due to a loss of pentoside, and an MS3 fragment at [M−H−132−162]^−^ due to a subsequent loss of hexoside. [20].

**Figure 5:**
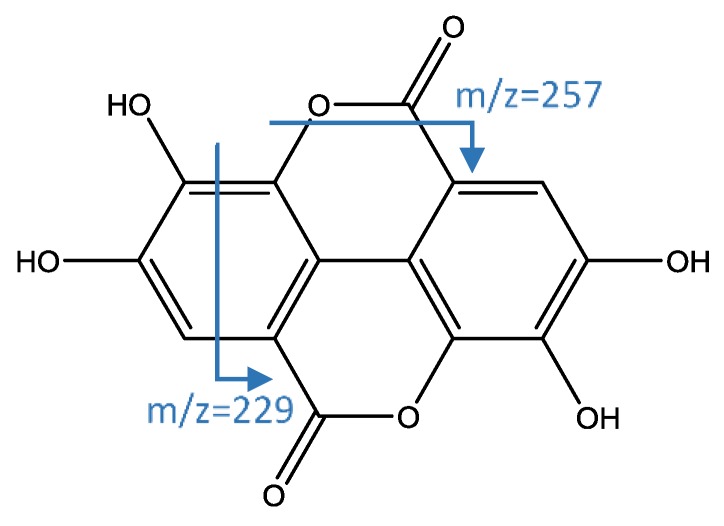
Structure and fragments of acid derivatives (35), (37) and (38).

Peaks 35, 37, and 38 (Figure 5) were identified as isomers of a derivate of syringic acid hexoside, which has an [M−H]^−^ ion at 403.1621 (C_18_H_27_O_10_) and successive fragments at *m*/*z* 241 (loss of hexoside) and 197 (aglycone of syringic acid) [43]. Peak 47 had [M−H]^−^ ion at 300.9995 (C_14_H_5_O_8_) that is coincident with ellagic acid, whose fragments at *m*/*z* 257 and 229 were previously reported [19,44].

#### 3.2.2. Other acids

**Figure 6:**
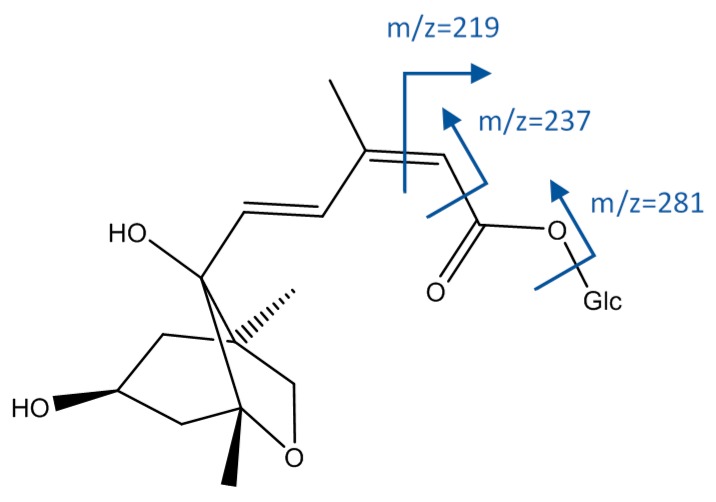
Structure and fragments of acid derivatives (1), (16), and (33).

As shown in Figure 6, peak 1, [M−H]^−^ = 191.0568, whose molecular formula was C_7_H_11_O_6_, agreed with quinic acid. Peaks 16 and 33 had an [M−H]^−^ ion at 443.1934 (C_21_H_31_O_10_), and fragments at *m*/*z* 425 (loss of water), 281 (loss of hexoside), 237 (α-cleavage to carbonyl group), and 219 (cleavage of double bond); so they were identified as isomers of dihydrophseic acid hexoside [45].

#### 3.2.3. Gallotanins

**Figure 7:**
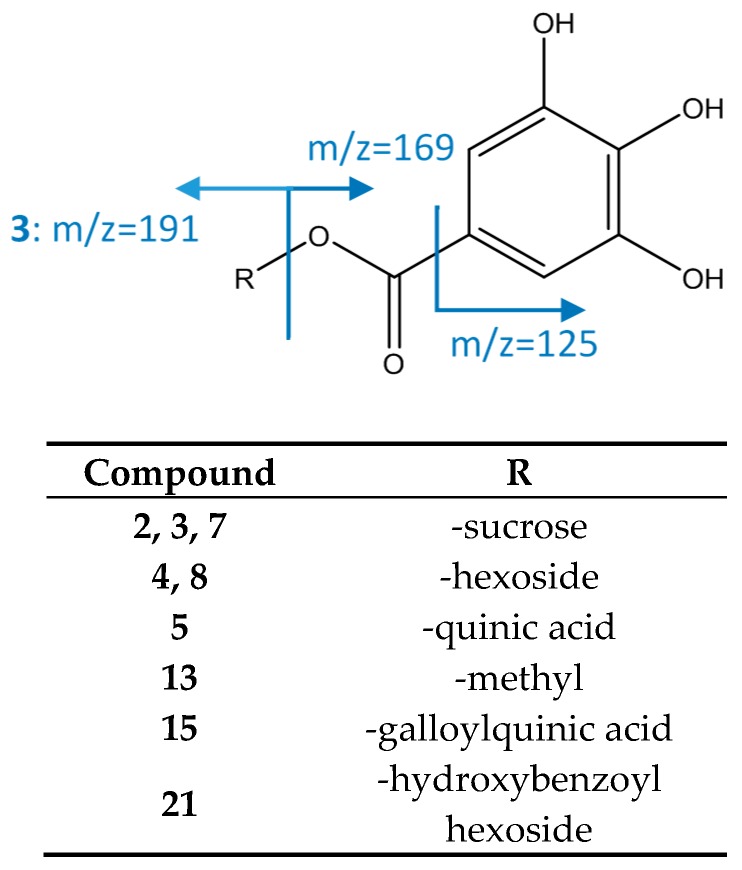
Structure and fragments of gallate derivatives.

As represented in Figure 7, compounds 4 and 8 (galloyl *O*-hexose isomers) show an [M−H]^−^ ion at *m*/*z* 331.0671 (C_13_H_15_O_10_), with a main fragment at *m*/*z* 169 corresponding to gallic acid. Peak 5 was identified as galloylquinic acid as it had an [M−H]^−^ ion at *m*/*z* 343.0667 (C_14_H_15_O_10_) and a fragment *m*/*z* 191 consistent with quinic acid due to loss of galloyl [19] Peaks 2, 3, and 7 with an [M−H]^−^ ion at *m*/*z* 493.1214 (C_19_H_25_O_15_) were assigned to galloyl *O*-sucrose due to the MS2 fragment at *m*/*z* 313, consistent with a loss of an *O*-hexoside, and MS3 fragment at *m*/*z* 169 corresponding to gallic acid (loss of second hexoside) [46].

Peak 13, [M−H]^−^ 357.0834 (C_15_H_17_O_10_), showed a main MS2 fragment at *m*/*z* 169 and an MS3 fragment at *m*/*z* 125, which is consistent with gallic acid, thus this peak was tentatively assigned to methyl gallate [19]. Peak 15 (di-*O*-galloyl quinic acid) had an [M−H]^−^ ion at *m*/*z* 495.0794 (C_21_H_19_O_14_), with a main fragment at *m*/*z* 343 due to the loss of a galloyl. A subsequent loss of quinic acid gives a fragment at *m*/*z* 169 [47]. Peak 21 was identified as hydroxybenzoyl galloyl hexoside due to its [M−H]^−^ ion at 451.0900 corresponding to a molecular formula of C_20_H_19_O_12_. The MS2 fragment at *m*/*z* 313 occurs due to a loss of hydroxybenzoyl, and MS3 at *m*/*z* 169 to subsequent loss of hexoside [20].

Peaks 11 and 20 had an [M−H]^−^ ion at 483.0799 (C_20_H_19_O_14_), with a main MS2 fragment at *m*/*z* 169, consistent with di-*O*-galloyl hexose isomers [19].

**Figure 8:**
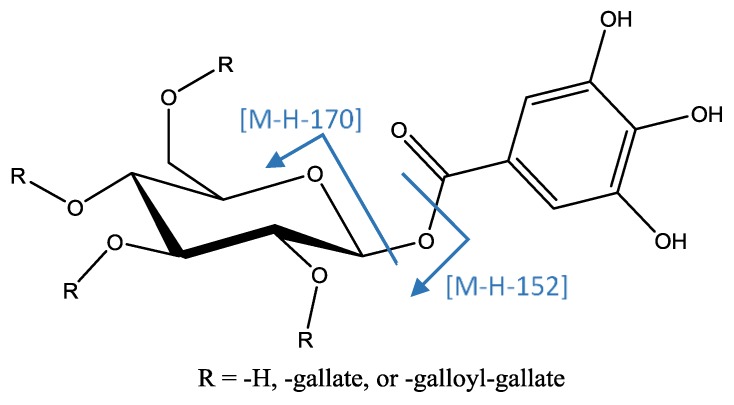
Structure and fragments of gallotannins.

A series of peaks (Figure 8) were identified as poly-*O*-galloyl hexoses due to their MS2 fragments at [M−H−170]^−^ (loss of O-galloyl) and [M−H−152]^−^ (loss of galloyl), and MS3 fragment at [M−H−170−152]^−^ due to the loss of a subsequent galloyl. This analysis allowed identification of peaks 19, 22, and 29 ([M−H]^−^ = 635.0888, C_27_H_23_O_18_) as tri-*O*-galloyl hexose; peaks 31, 32, 40, 41, 44, and 45 ([M−H]^−^ = 787.1008, C_34_H_27_O_22_) as tetra-*O*-galloyl hexose; and peak 51 ([M−H]^−^ = 939.1132, C_41_H_31_O_26_) as penta-*O*-galloyl hexose [20].

Compounds with more units of gallate in their structures show an MS2 fragment at [M−H−152]^−^ due to loss of galloyl, and loss of a second galloyl gave an MS3 fragment at [M−H−152−152]^−^. Therefore it was possible to identify peaks 54, 55, and 56 ([M−H]^−^ = 1091.1238, C_48_H_35_O_30_) as hexa-*O*-galloyl hexose isomers; peaks 57, 58, and 59 ([M−H]^−^ = 1234.1351, C_55_H_39_O_34_) as hepta-*O*-galloyl hexose isomers; peaks 60 and 61 ([M−H]^−^ = 1395.1466, C_62_H_43_O_38_) as octa-*O*-galloyl hexose isomers; peaks 63 and 64 ([M−H]^−^ = 1547.1576, C_69_H_47_O_42_) as nona-*O*-galloyl hexose isomers; peaks 65 and 66 ([M−H]^−^ = 1699.1690, C_76_H_51_O_46_) as deca-*O*-galloyl hexose isomers; and peaks 67, 69, and 70 ([M−H]^−^ = 1851.1819, C_83_H_55_O_50_) as isomers of undeca-*O*-galloyl hexose isomers [15].

#### 3.2.4. Hydroxybenzophenones

**Figure 9:**
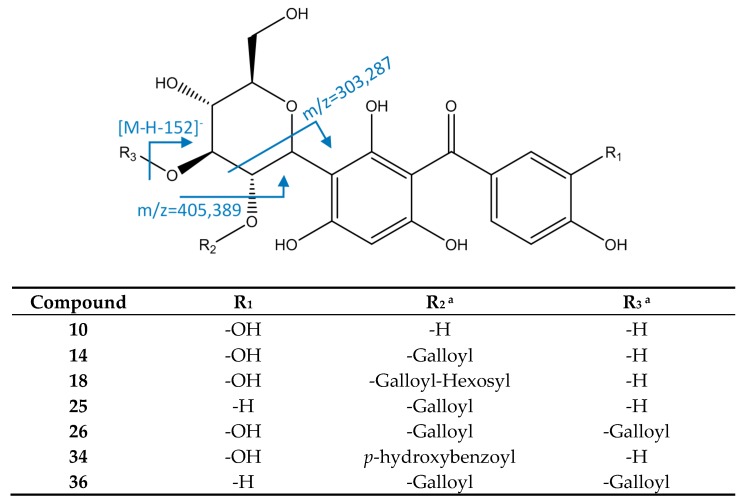
Structure and fragments of hydroxybenzophenones ^a^ Positions of substituents may vary among the hexoside ring.

Maclurin 3-C-hexoside derivates (Figure 9) were identified by their molecular formula and characteristic fragment at *m*/*z* 303 due to retro Diels–Alder cleavage of hexoside. Thus, peak 10 ([M−H]^−^ = 423.0943, C_19_H_19_O_11_) was identified as maclurin 3-C-hexoside. Peak 14 ([M−H]^−^ = 575.1047, C_26_H_23_O_15_) had main fragments at *m*/*z* 423 (loss of galloyl) and 303, so it was identified as maclurin 3-C-(2-O-galloyl)-hexoside. Peak 18 ([M−H]^−^ = 737.1588, C_32_H_33_O_20_) showed a main MS2 fragment at *m*/*z* 575 due to a loss of hexoside, and MS3 fragments at *m*/*z* 423 and 303 as the previous peak, so it was identified as maclurin 3-C-(2-O-hexosyl-galloyl)-hexoside. Peak 26 ([M−H]^−^ = 727.1166, C_33_H_27_O_19_), identified as maclurin 3-C-(2,3-di-O-galloyl)-hexoside, had an MS2 fragment at *m*/*z* 575 due to the loss of one galloyl, and main MS3 fragments at *m*/*z* 485 (cleavage of hexoside) and 405 (loss of O-galloyl). Peak 34 ([M−H]^−^ = 543.1149, C_26_H_23_O_13_) had a main fragment at *m*/*z* 285 due to the loss of water of a fragment at *m*/*z* 303, coincident with reports for maclurin-3-C-(p-hydroxybenzoyl)-hexoside [19,48].

Peak 25 ([M−H]^−^ = 559.1101, C_26_H_23_O_14_) was identified as iriflophenone 3-C-(2-O-galloyl)- hexoside, due to its MS2 fragment at *m*/*z* 407 ([M−H−152]^−^, loss of galloyl) and MS3 fragment at *m*/*z* 287 (hexoside cleavage). Peak 36 ([M−H]^−^ = 711.1216, C_33_H_27_O_18_) had an MS2 fragment at *m*/*z* 559 due to a loss of a galloyl, and an MS3 fragment at 387 (loss of O-galloyl), so it was assigned to iriflophenone 3-C-(2,3-di-O-galloyl)-hexoside [49].

#### 3.2.5. Xanthonoids

**Figure 10:**
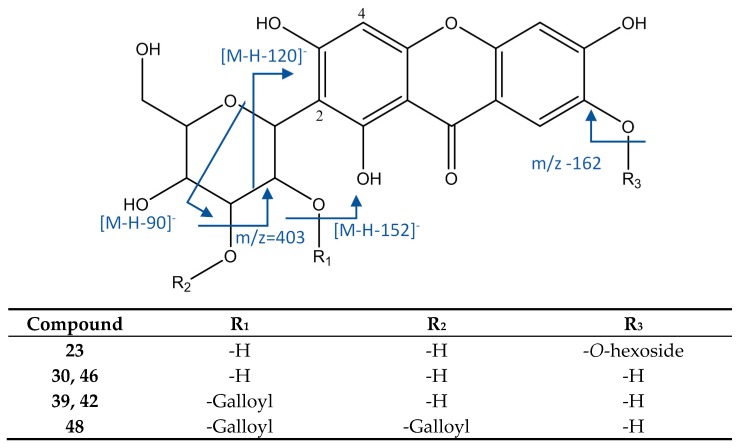
Structure and fragments of xanthonoids.

As represented in Figure 10, peak 23 had an [M−H]^−^ ion at 583.1311, with a molecular formula of C_25_H_27_O_16_. It showed MS2 fragments at *m*/*z* 565 (loss of water), 493 [M−H−90]^−^, and 463 [M−H−120]^−^ (both due to cleavage of C-hexoside). The main MS3 fragment at *m*/*z* 331 [M−H−90-162]^−^ occurred by the loss of a O-hexoside. Thus, this peak was identified as mangiferin O-hexoside [50].

Peaks 30 and 46 were identified as isomers mangiferin and isomangiferin as they had an [M−H]^−^ ion at 421.0787 (C_19_H_17_O_11_) and fragments at *m*/*z* 331 [M−H−90]^−^ and 301 [M−H−120]^−^, both of them due to cleavage of glicoside. Peaks 39 and 42 showed an [M−H]^−^ ion at 573.0891 (C_26_H_21_O_15_), with an MS2 fragment at *m*/*z* 421 [M−H−152]^−^ and the same MS3 fragments as mangiferin, thus they were identified as the isomers maguiferin O-gallate and isomangiferin O-gallate [16]. Peak 48 ([M−H]^−^ = 725.1004, C_33_H_25_O_19_) had an MS2 fragment at *m*/*z* 573 ([M−H−152]^−^, loss of galloyl) and MS3 fragments at *m*/*z* 421 (loss of second galloyl) and 403 (loss of O-galloyl), so it was assigned to mangiferin-di-O-gallate [48]. 

#### 3.2.6. Flavonoids

**Figure 11:**
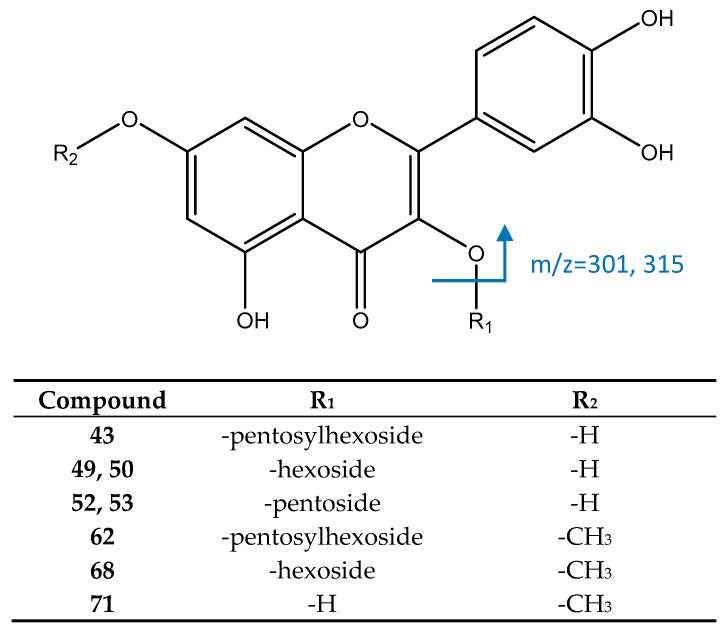
Structure and fragments of flavonoids.

Peak 43, [M−H]^−^ = 595.1303 (C_26_H_27_O_16_) showed (Figure 11) fragments at *m*/*z* 300 and 301, coincident with a quercetin aglycone. Thus, this peak was identified as quercetin-3-*O*-pentosylhexoside. Peaks 49 and 50 were identified as isomers of quercetin 3-*O*-hexoside, due to their [M−H]^−^ ion at 463.0893 (C_21_H_19_O_12_) and the fragment at *m*/*z* 301 ([M−H−162]^−^, loss of hexoside). Similarly, peaks 52 and 53 ([M−H]^−^ = 433.0786, C_20_H_17_O_11_) had the same fragment at *m*/*z* 301 [M−H−132]^−^ due to the loss of a pentoside, so they were identified as isomers of quercetin 3-*O*-pentoside [19].

Peak 62 had an [M−H]^−^ ion at 609.1467, consistent with the molecular formula of C_27_H_29_O_16_. Its main MS2 fragments at *m*/*z* 314 and 315 suggest the presence of a rhamnetin aglycone, so it was assigned to rhamnetin *O*-pentosylhexoside [51]. Peak 68, with [M−H]^−^ = 447.1054, C_22_H_21_O_12_, was identified as rhamnetin *O*-hexoside, due to its MS2 fragment at *m*/*z* 315 (loss of hexoside) [19] while peak 71 ([M−H]^−^ = 315.0513, C_16_H_11_O_7_) was identified as the aglycone rhamnetin, with a main fragment at *m*/*z* 271 coincident with previous reports [52,53].

Regarding the total number of polyphenols in *M. indica* Keitt and T. Atkins cultivars, 149 compounds were found, comprising 12 xanthonoids, 32 phenolic acids and derivatives, 10 hydroxybenzophenones, 10 flavonoids, and 85 gallates and gallotannins. T. Atkins skins have a greater number of compounds than Keitt skins, which exhibit the highest number of gallates and galloltannins with 32 different compounds. In respect to compound diversity, gallotannins are the most recurrent group of polyphenols in both Keitt and T. Atkins skin samples and in Keitt flesh while phenolic acids are the most abundant group in T. Atkins flesh. Xanthonoids are present in all skin and flesh samples and the other two subfamilies differ in their occurrence. For instance, flavonoids are more abundant in T. Atkins skin while hydroxybenzophenones are found only in the skins of both cultivars.

When comparing reports from the literature, our findings coincide with previous reports on Keitt and T. Atkins cultivars from Brazil and Spain [15,17,20,54] showing that skins have a higher number and diversity of polyphenols than flesh and also that Keitt skin and flesh show a similar phenolic profile while the T. Atkins cultivar shows a different profile, with very low diversity for flesh. On the other hand, our results for the Keitt cultivar, which has been given more attention in the literature, indicate a higher number and diversity of compounds than those reported for cultivars from China, Spain, and the USA [13,19,21], particularly in xanthonoids and gallate and gallotannins, with similar results to other reports on cultivars from Spain [20,54].

On the other hand, when comparing the reports for other cultivars in the literature, our results for Keitt and T. Atkins skin cultivars from Costa Rica show a greater number and diversity of polyphenols than the findings on 18 cultivars from Australia, Kenya, Peru, and Thailand [17], as well as from Brazil [55], China [21], and Spain [19], and similar results to the Sensation cultivar from Spain [54]. In the case of flesh, T. Atkins results are similar to cultivars from Mexico and Haiti [8] and higher than those for the Haden cultivar from Brazil [55], while Keitt flesh shows greater and more diverse results than for the other nine different cultivars from Peru and Thailand [17]; as well as from Brazil [55], Mexico, and Haiti [8]; and similar results to the Sensation cultivar from Spain [54].

Finally, our findings for Costa Rican cultivars indicate an important number of gallotannin oligomers of a higher polymerization degree in both Keitt and T. Atkins skins as well as in Keitt flesh; for instance, galloyl hexose decamers and undecamers that are reported for the first time for these two cultivars. These results are of special interest due to reports showing an enhancement of anti-inflammatory and anticancer activities, with gallotannins’ higher degree of polymerization, for instance, showing better results than dimers or the galloyl hexose monomer [22].

### 3.3. Antioxidant Activity

The DPPH and ORAC values obtained are summarized in Table 3. Regarding DPPH, one-way ANOVA with a Tukey post hoc as the statistical test shows a significant difference (*p* < 0.05) between the results for polyphenolic enriched extracts of skin and flesh. For instance, skins show a higher antioxidant activity, with an average of 10.95 μg extract/mL while the flesh average is 20.15 μg extract/mL. At the individual level, T. Atkins skin presents the greater antioxidant value (IC_50_ = 9.97 μg extract/mL), followed by Keitt skin (IC_50_ = 11.93 μg extract/m). Both are higher than findings for the skin of cultivars from Chile reporting a value of IC_50_ = 46.39 μg extract/mL for T. Atkins cultivar and IC_50_ = 32.23 μg extract/mL for Pica cultivar [34]. This trend is also found when comparing flesh from these cultivars, where IC_50_ = 122.22 μg extract/mL and IC_50_ = 73.76 μg extract/mL were reported for T. Atkins and Pica mangoes [34], lower than our results for Keitt (IC_50_ = 17.78 μg extract/mL) and T. Atkins (IC_50_ = 22.51 μg extract/mL) cultivars. On the other hand, when comparing our DPPH values expressed as mmol TE/g extract (in respect to Trolox IC_50_ = 5.62 μg/mL) with reports in the literature, it is observed that Keitt and T. Atkins skin values fit in the range between α-tocopherol and enju extract (0.42–0.76 mmol TE/g extract), which are existing antioxidant food additives [56].

In respect to ORAC evaluation, the results also show significant differences (ANOVA, *p* < 0.05) between skin and flesh extracts. In fact, skins exhibit a higher antioxidant activity, with an average of 9.66 mmol TE/g extract, while the flesh extract average is 4.68 mmol TE/g extract. At the individual level, T. Atkins skin also shows the highest antioxidant value (11.02 mmol Trolox equivalents/g extract), followed by Keith skin (8.30 mmol TE/g extract), while the lowest value is shown by T. Atkins flesh (3.56 mmol TE/g extract). A previous study on the flesh of different cultivars from Spain reported an ORAC value of 156.6 μmol TE/100 g fresh weight for T. Atkins cultivar and higher values for Francis and Ataulfo cultivars (225.8 and 326.6 μmol TE/100 g fresh weight, respectively) [8], all of which are lower than our results for Keitt flesh (357.1 μmol TE/100 g fresh weight) and T. Atkins flesh (606.4 mmol TE/100 g fresh weight).

Finally, a correlation analysis was carried out between total polyphenolic contents (TPC) values (Table 1) and the DPPH and ORAC results. The findings indicate a significant positive correlation (*p* < 0.05) between both DPPH (mmol TE/g extract) and ORAC antioxidant findings (*r* = 0.989), and between TPC and ORAC values (*r* = 0.910), and a significant negative correlation (*p* < 0.05) was found between the TPC results and DPPH values (*r* = −0.960). Therefore, our results align with previous studies reporting a correlation between the total polyphenolic contents and antioxidant activities of *Mangifera indica* cultivars [54].

### 3.4. Cytotoxicity

**Figure 12:**
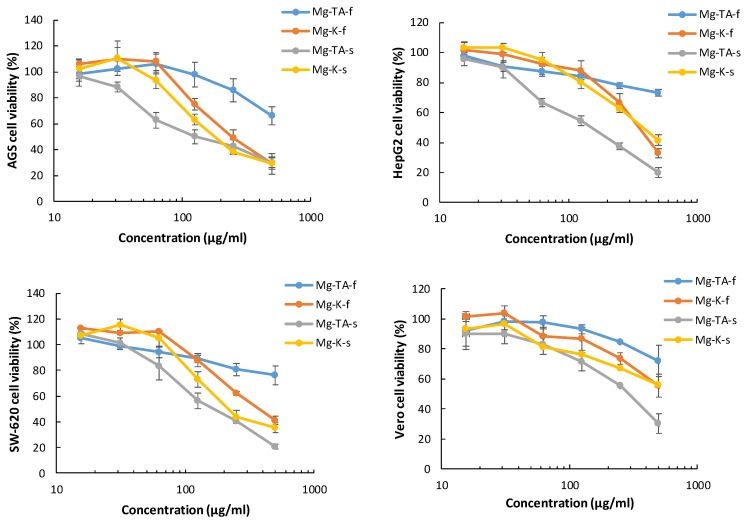
Cytotoxicity dose–response curves of *M. indica* extracts on AGS, HepG2, and SW620 adenocarcinoma cell lines and Vero normal cell lines. Results are presented as mean ± SD of three independent experiments. Mg-TA-f (T. Atkins cultivar flesh), Mg-K-f (Keitt cultivar flesh), Mg-TA-s (T. Atkins cultivar skin), and Mg-K-s (Keitt cultivar skin).

Table 4 and Figure 12 summarize the IC_50_ values for the cytotoxic effect of *M. indica* extracts on different human carcinoma cells, namely AGS, HepG2, and SW620 cell lines, all related to the digestive tract. The development of these types of cancers has been associated to a lower consumption of vegetables and fruits [57], particularly 60% of stomach cancer and 43% of colon cancer are attributed to deficient consumption of vegetables [58]. For this reason, it is interesting to evaluate the cytotoxicity activity of fruit phytochemical-enriched extracts on these tumoral cells. 

The IC_50_ values shown in Table 4 suggest that a better cytotoxic effect is displayed by the extracts obtained from skin than the extracts obtained from flesh. This difference is statistically significant for all cell lines in the T. Atkins cultivar and for SW-620 cells in the Keitt cultivar. The better results of the skin extracts are in agreement with results previously described for other cultivars [35,59,60]. Also, publications that compared extracts from different parts of the mango highlight the potential of skin and kernel extracts in the cytotoxic activity of different cell lines [61,62].

As shown in Table 4, in all assessed samples, the comparison of the cytotoxic effect between the different types of carcinoma cell lines showed better IC_50_ values against the gastric cells (AGS) than the hepatic (HepG2) and colon (SW-620) cell lines. Few reports of cytotoxic effects are available for gastric adenocarcinoma cell lines; for instance, for skin extracts of the Irwi mango cultivar, a dose-dependent effect from 125 to 1000 μg/mL was described on AGS cells [35] and a low cytotoxic effect on Kato-III cells using an ethanolic leaf extract of Okrong mango (IC_50_ > 200 μg/mL) [63]. Our results show better cytotoxic activity in ranges of 15 to 500 μg/mL, with an IC_50_ of 138 ± 8 μg/mL for Tommy Atkins skin and 197 ± 16 μg/mL for Keith skin cultivars. 

Table 4 also shows a significantly better cytotoxic effect on the hepatocellular carcinoma cells (HepG2) for the T. Atkins cultivar compared to Keitt mango. In fact, the IC_50_ value assessed for T. Atkins skin is 164 ± 13 μg/mL, which is similar to results reported for the Irwin cultivar, which caused a decrease of 50% viability in concentrations between 125 and 250 μg/mL when incubated with HepG2 cells [35]. However, skin extracts of another mango species, *M. pajang*, showed better cytotoxic activity for HepG2 (IC_50_ = 36.5 μg/mL) than *M. indica* varieties [61].

On the other hand, the cytotoxic effect of *M. indica* cultivar skin extracts on colon carcinoma (SW-620) shows an IC_50_ of 175 ± 7 μg/mL for T. Atkins skin and 223 ± 24 μg/mL for the Keitt cultivar (Table 4). Previous reports for other varieties of *M. indica* cited IC_50_ values over 200 μg/mL for pulp extract [64] in similar colon carcinoma cell lines. In addition to the cytotoxic effect, the extracts used in this study showed a significant difference (*p* < 0.05) between the IC_50_ of the control non-tumoral cell line (Vero) and the tumoral cell lines. This kind of selectivity is a desirable characteristic for any compound with chemotherapy potential [65,66].

Finally, the results of the correlation analysis of cytotoxic activities (Table 4) for each cell line with the abundance of polyphenolic compounds in each sample (Table 2) show a significant negative correlation between cytotoxic effects and the number of polyphenols for all cell lines, AGS (*r* = −0.984), HepG2 (*r* = −0.974), and SW620 (*r* = 0.983), at *p* < 0.05. In fact, T. Atkins skin constitutes the sample with more polyphenolic compounds and with the best cytotoxic activity, followed by the skin of the Keitt cultivar. Specific correlations between the cytotoxic activity and the presence of each type of polyphenolic compounds identified were calculated and it was observed that for AGS and SW 620 cell lines, the cytotoxic activity showed a particularly high significant negative correlation with gallotannins (*r* = −0.977 and *r* = −0.940, respectively), followed by a significant negative correlation of the SW620 cytotoxicity results with xanthonoids (*r* = −0.880). In turn, cytotoxic effects on the HepG2 cell line had the best significant negative correlation with xanthonoids (*r* = −0.921). These specific correlation values between the types of phenolic compounds and cytotoxic activity suggest that gallotannins and xanthonoids play an important role in the toxicity against cancer cells. In fact, previous reports suggest that these two classes of compounds seem to be strong determinants of the anti-tumor activity of mango extracts [14].

One of the main xanthonoids present in the skin extracts of our study is mangiferin and its isomeric forms (Table 2). These compounds have been described in the literature as promising anticancer polyphenols [11,67,68]. The ability of mangiferin to inhibit cancer cells is achieved through several molecular targets, however, one of the important mechanisms is associated with induction of apoptosis [68,69]. Although, besides apoptosis induction, other mechanisms of cell cytotoxicity have been postulated for mangiferin [11], such as cell cycle arrest [70] and a decrease in matrix metalloproteinase activities and reversal of the epithelial–mesenchymal transition [71]. 

Concerning gallates or gallotannins identified in mango extracts, in vitro studies have shown strong cytotoxic activity; for instance, inhibitions of 55% to 75% in the proliferation of breast, liver, and leukemia cancer cell lines treated with 40 to 80 μg/mL of extracts from Chinese cultivars [6]. The main compounds detected in these mango extracts correspond to pentagalloyl hexose to nonagalloyl hexose isomers. Also, these authors tested penta-galloyl hexoside and gallic acid to treat the same cell lines and confirm the potential role of these compounds as antiproliferatives. Another study on Keitt extract containing galloyl hexosides ranging from pentagalloyl hexose to nonagalloyl hexose inhibited 90% of breast cancer cell lines, with a concentration of 10 μg/mL evidencing strong activity [19].

Table 2 shows that skin extracts from mango contain a high number of gallotannins with different degrees of polymerization, ranging from monogalloyl hexose to undeca-galloyl hexoses. Studies of gallotannins from red maple species in colon and breast cancer cells showed an association between the higher number of galloyl groups in the gallotannins and better anticancer activity [22]. Particularly, the penta galloyl glucoside from fruits, such as maple, gallnuts, and oak, and medicinal herbs (*Galla rhois, Rhus chineensis, Paeonia suffruticosa*) has been widely associated to anticancer effects in prostate, breast, glioma, hepatocellular, and colorectal carcinoma [72,73,74]. The suggested mechanisms associated to the cytotoxicity involve induction of apoptosis through an increase of Bax/Bcl-2 protein levels, cell cycle arrest in S-phase, and the inhibition of NF-κB activation, with the consequent downregulation of inflammatory cytokines [73].

Even though our results and the cited previous reports suggest that gallates and xanthonoids seem to be strong determinants of the anti-tumor activity, the mechanism of cell cytotoxicity has to be determined and the target molecules of the *M. indica* extract remain to be identified. Also, despite our results indicating a correlation with the presence of xanthonoids and gallates and the cytotoxic activity in tumoral cell lines, these compounds are probably not the only factors responsible for the observed biological effects. The contribution of other components present in the extracts should be clarified because reports in the literature of similar anti-cancer properties are available for phenolic acids and flavonoids [75,76] and hydroxybenzophenones [77].

## 4. Conclusions

The analysis of phenolic-enriched extracts of Keitt and T. Atkins, the main commercial cultivars of *M. indica* in Costa Rica, using UPLC-DAD-ESI-MS techniques identified a total of 149 compounds, 82 of them in the Keitt cultivar, including 54 different gallotannins, which demonstrates the potential value of this fruit with a greater and more diverse number of compounds than cultivars from different countries and similar to previous important results reported for Keitt fruits from Spain [20,50]. Besides, our results for T. Atkins skin showing 59 compounds, including 30 gallotannins, constitutes the first report of such a high number and diversity of polyphenolic compounds for this cultivar. On the other hand, the TPC, DPPH, and ORAC antioxidant capacity showed high significant correlations (*p* < 0.05), with Keitt and T. Atkins skins exhibiting the highest values. Further, cytotoxicity results were also better for skin extracts in all three adenocarcinoma cell lines studied. For AGS and SW 620 cell lines, cytotoxicity activity showed a particularly high significant negative correlation with gallotannins (*r* = −0.977 and *r* = −0.940, respectively), while for the HepG2 cell line, the highest significant negative correlation was found with xanthonoid compounds (*r* = −0.921). These results and the presence of diverse xanthonoids and numerous gallotannins of a high polymerization degree, such as decamers (decagalloyl hexoses) and undecamers (undecagalloyl hexoses), which are reported for the first time for these *M. indica* cultivars, suggest the potential of these extracts for further studies. For instance, xanthonoids have been linked with anti-inflammatory and anticancer activities, and gallotannins of a higher degree of polymerization have been found to enhance such properties [22]; hence, structure–activity relationship studies would contribute to increase the knowledge on the fruits as a source of dietary compounds and bioactivities associated with potential health benefits.

## Figures and Tables

**Table 1:** Extraction yield and total phenolic content.

Sample	Lyophilization Yield (g/100 g) ^1^	Extraction Yield (g/100 g) ^2^	Total Phenolic Content (TPC) (mg/g) ^3,4,5^
Keitt			
Skin	20.8	2.77	698.65 ± 0.47 ^a^
Flesh	16.9	0.69	291.14 ± 1.19 ^b^
T. Atkins			
Skin	21.5	2.75	644.17 ± 5.79 ^c^
Flesh	17.6	0.57	162.67 ± 1.46 ^d^

^1^ g of dry material/100 g of fresh weight ^2^ g of phenolic enriched extract/100 g of dry material ^3^ mg of gallic acid equivalents (GAE)/g extract. ^4^ Values are expressed as mean ± standard deviation (S.D.) ^5^ Different superscript letters in the column indicate differences are significant at *p* < 0.05.

**Table 2:** Profile of phenolic compounds identified by UPLC-DAD-ESI-TQ-MS analysis for mangoes Keitt and T. Atkins samples.

Peak	Identification	Rt (min)	[M−H]^−^	Molecular Formula	Error (ppm)	MS2 Fragments	MS3 Fragments	Keith Skin	Keit Flesh	T. A. Skin	T. A. Flesh
*Phenolic Acis*											
6	Hydroxybenzoic acid hexoside (isomer I of II)	4.43	299.078	C_13_H_15_O_8_	6.31	[299]: 137(100)	[299→137]: 93(100)		x		
9	Hydroxybenzoic acid hexoside (isomer II of II)	5.80	299.0775	C_13_H_15_O_8_	4.37	[299]: 137(100). 179(75). 239(79)	[299→137]: 93(100)	x	x		
12	5-hydroxyferuloyl hexoside	7.64	371.0993	C_16_H_19_O_10_	5.38	[371]: 209(90). 233(100)	[371→233]: 191(100), 205(89)			x	
17	Ferulic acid	10.47	193.0515	C_10_H_9_O_4_	10.07	[193]: 149(100). 178(73)	[193→149]: 134(100)		x		
24	Sinapic acid	12.03	223.0618	C_11_H_11_O_5_	7.40	[223]: 164(17). 179(32). 208(100)	[223→208]: 164(100)		x	x	
27	Sinapic acid *O*-pentosyl-hexoside	13.27	517.2304	C_24_H_37_O_12_	4.75	[517]: 205(93). 385(100)	[517→385]: 153(100), 205(87), 223(90)	x	x	x	x
28	Dihydrosinapic acid *O*-pentosyl-hexoside	13.47	519.2462	C_24_H_39_O_12_	5.04	[519]: 387(100)	[519→387]: 161(100), 225(63)	x	x	x	x
35	Syringic acid hexoside derivative (isomer I of III)	14.75	403.1621	C_18_H_27_O_10_	5.55	[403]: 241(100)	[403→241]: 197(100)		x		
37	Syringic acid hexoside derivative (isomer II of III)	15.18	403.1621	C_18_H_27_O_10_	5.62	[403]: 241(100)	[403→241]: 197(100)	x	x		
38	Syringic acid hexoside derivative (isomer III of III)	15.47	403.1618	C_18_H_27_O_10_	4.70	[403]: 241(100)	[403→241]: 197(100)		x		
47	Ellagic acid	16.55	300.9999	C_14_H_5_O_8_	6.60	[301]: 229(52). 257(100)		x		x	x
*Other acids*											
1	Quinic acid	1.58	191.0568	C_7_H_11_O_6_	9.24	[191]: 85(69). 93(57). 127(100). 173(83)		x		x	
16	Dihydrophaseic acid hexoside (isomer I of II)	9.99	443.1934	C_21_H_31_O_10_	5.00	[443]: 161(90). 189(48). 219(83). 237(100). 281(44). 425(35)	[443→237]: 219(100)	x	x	x	x
33	Dihydrophaseic acid hexoside (isomer II of II)	14.59	443.1940	C_21_H_31_O_10_	6.45	[443]: 189(51). 219(52). 237(87). 263(100). 281(34). 399(33). 425(97)		x	x	x	x
*Gallates and Gallotannins*											
2	Galloyl dihexoside (isomer I of III)	1.79	493.1210	C_19_H_25_O_15_	4.47	[493]: 313(100)	[493→313]: 169(100), 223(52)			x	
3	Galloyl dihexoside (isomer II of III)	2.35	493.1213	C_19_H_25_O_15_	4.98	[493]: 313(100)	[493→313]: 169(100), 223(52)			x	
4	Galloyl *O*-hexose (isomer I of II)	2.67	331.0671	C_13_H_15_O_10_	3.34	[331]: 169(100), 211(31), 271(79)	[331→169]: 125(100)	x	x	x	x
5	Galloylquinic acid	3.81	343.0667	C_14_H_15_O_10_	2.24	[343]: 191(100)	[343→191]: 85(77), 93(61), 111(35), 126(100), 173(79)	x	x	x	
7	Galloyl *O*-dihexoside (isomer III of III)	4.86	493.1214	C_19_H_25_O_15_	3.09	[493]: 313(100)	[493→313]: 169(100), 223(52)	x	x	x	
8	Galloyl *O*-hexose (isomer II of II)	5.23	331.0674	C_13_H_15_O_10_	4.43	[331]: 169(100)	[331→169]: 125(100)	x	x	x	
11	Di-*O*-galloyl hexose (isomer I of II)	7.28	483.0799	C_20_H_19_O_14_	6.21	[483]: 169(100)	[483→169]: 125(100)	x	x	x	
13	Methyl-gallate isomer	8.63	357.0834	C_15_H_17_O_10_	4.89	[357]: 169(100)			x	x	
15	Di-*O*-galloyl quinic acid	9.74	495.0794	C_21_H_19_O_14_	4.28	[495]: 343(100)	[495→343]: 169(100)	x		x	
19	Tri-*O*-galloyl hexose (isomer I of III)	10.82	635.08978	C_27_H_23_O_18_	2.98	[635]: 465(100), 483(95)	[635→465]: 168(59), 295(31), 313(87), 421(100)	x		x	
20	Di-*O*-galloyl hexose (isomer II of II)	10.94	483.0792	C_20_H_19_O_14_	2.26	[483]: 331(100)	[483→331]: 169(100)	x		x	
21	Hydroxybenzoyl galloyl hexoside	11.35	451.0900	C_20_H_19_O_12_	6.42	[451]: 313(100)	[451→313]: 169(100)		x		
22	Tri-*O*-galloyl hexose (isomer II of III)	11.38	635.0888	C_27_H_23_O_18_	1.35	[635]: 465(100), 483(95)	[635→465]: 168(59), 295(31), 313(87), 421(100)	x		x	
29	Tri-*O*-galloyl hexose (isomer III of III)	13.68	635.08942	C_27_H_23_O_18_	2.41	[635]: 465(100), 483(95)	[635→465]: 168(59), 295(31), 313(87), 421(100)	x		x	
31	Tetra-*O*-galloyl hexose (isomer I of VI)	14.23	787.1008	C_34_H_27_O_22_	1.98	[787]: 635(100)	[787→635]: 423(77), 465(100), 483(99)	x	x	x	
32	Tetra-*O*-galloyl hexose (isomer II of VI)	14.37	787.1013	C_34_H_27_O_22_	3.06	[787]: 635(100)	[787→635]: 423(77), 465(100), 483(99)	x		x	
40	Tetra-*O*-galloyl hexose (isomer III of VI)	15.61	787.1015	C_34_H_27_O_22_	2.65	[787]: 617(100), 635(53)	[787→617]: 447(32), 465(100)	x		x	
41	Tetra-*O*-galloyl hexose (isomer IV of VI)	15.73	787.1017	C_34_H_27_O_22_	3.60	[787]: 617(25), 635(100)	[787→635]: 423(74), 465(76), 483(100)	x	x	x	
44	Tetra-*O*-galloyl hexose (isomer V of VI)	15.98	787.1016	C_34_H_27_O_22_	3.52	[787]: 617(100)	[787→635]: 403(62), 447(65), 465(100)	x	x	x	
45	Tetra-*O*-galloyl hexose (isomer VI of VI)	16.32	787.1010	C_34_H_27_O_22_	2.75	[787]: 635(100)	[787→635]: 423(77), 465(100), 483(99)	x		x	
51	Penta-*O*-galloyl hexose	17.27	939.1132	C_41_H_31_O_26_	−2.68	[939]: 769(100)	[939→769]: 599(31), 601(32), 617(100)	x	x	x	
54	Hexa-*O*-galloyl hexose (isomer I of III)	18.07	1091.1238	C_48_H_35_O_30_	0.6	[1091]: 939(100)	[1091→939]: 769(100)	x	x	x	
55	Hexa-*O*-galloyl hexose (isomer II of III)	18.37	1091.1227	C_48_H_35_O_30_	−0.41	[1091]: 939(100)	[1091→939]: 769(100)	x	x	x	
56	Hexa-*O*-galloyl hexose (isomer III of III)	18.52	1091.1235	C_48_H_35_O_30_	0.38	[1091]: 939(100)	[1091→939]: 769(100)	x		x	
57	Hepta-*O*-galloyl hexose (isomer I of III)	18.80	1243.1351	C_55_H_39_O_34_	−0.10	[1243]: 939(48), 1091(100)	[1243→1091]: 939(100)	x	x	x	
58	Hepta-*O*-galloyl hexose (isomer II of III)	18.94	1243.1349	C_55_H_39_O_34_	−2.18	[1243]: 939(48), 1091(100)	[1243→1091]: 939(100)	x	x	x	
59	Hepta-*O*-galloyl hexose (isomer III of II)	19.06	1243.1352	C_55_H_39_O_34_	2.74	[1243]: 939(56), 1091(100)	[1243→1091]: 939(100)	x		x	
60	Octa-*O*-galloyl hexose (isomer I of II)	19.39	1395.1466	C_62_H_43_O_38_	2.81	[1395]: 1243(100), 1244(58)		x	x	x	
61	Octa-*O*-galloyl hexose (isomer II of II)	19.60	1395.1464	C_62_H_43_O_38_	2.64	[1395]: 1243(100), 1244(41)		x	x	x	
63	Nona-*O*-galloyl hexose	20.11	1547.1576	C_69_H_47_O_42_	2.55	[1547]: 1395(100), 1396(62)		x	x		
64	Nona-*O*-galloyl hexose	20.44	1547.1603	C_69_H_47_O_42_	4.29	[1547]: 1395(100), 1396(62)		x		x	
65	Deca-*O*-galloyl hexose	20.60	1699.1690	C_76_H_51_O_46_	2.56	[1699]: 1547(100), 1548(53)		x	x		
66	Deca-*O*-galloyl hexose	20.80	1699.1724	C_76_H_51_O_46_	4.57	[1699]: 1547(100), 1548(53)		x		x	
67	Undeca-*O*-galloyl hexose (isomer I of III)	20.94	1851.1819	C_83_H_55_O_50_	3.42	[1851]: 1547(33), 1699(100), 1700(90)		x	x		
69	Undeca-*O*-galloyl hexose (isomer II of III)	21.28	1851.1805	C_83_H_55_O_50_	2.69	[1851]: 1395(43), 1547(47), 1699(100), 1700(81)		x	x		
70	Undeca-*O*-galloyl hexose (isomer III of III)	21.53	1851.1803	C_83_H_55_O_50_	2.56	[1851]: 1395(100), 1547(76), 1699(99), 1700(85)		x	x		
*Xanthonoids*											
10	Maclurin C-hexoside	6.62	423.0943	C_19_H_19_O_11_	5.94	[423]: 303(100)	[423→303]: 193(100)			x	
14	Maclurin 3-C-(2-*O*-galloyl)-hexoside	9.35	575.1047	C_26_H_23_O_15_	2.67	[575]: 285(85), 303(100), 313(43), 423(70), 465(31)	[575→303]: 193(100)	x		x	
18	Maclurin-3-C-(2-*O*-hexosyl-galloyl)-hexoside	10.69	737.1588	C_32_H_33_O_20_	2.78	[737]: 575(100)	[737→575]: 285(89), 303(100), 313(44), 423(80)			x	
25	Iriflophenone 3-C-(2-*O*-galloyl)-hexoside	12.31	559.1101	C_26_H_23_O_14_	3.36	[559]: 287(31), 407(100)	[559→407]: 287(100)	x		x	
26	Maclurin 3-C-(2.3-di-*O*-galloyl)- hexoside	12.82	727.1166	C_33_H_27_O_19_	3.49	[727]: 575(100)	[727→ 575]: 315(39), 369(38), 405(100), 439(56), 465(37), 485(78)	x		x	
34	Maclurin-3-C-(*p*-hydroxybenzoyl)- hexoside	14.63	543.1149	C_26_H_23_O_13_	5.91	[543]: 285(100)	[543→285]: 175(100)			x	
36	Iriflophenone 3-C-(di-*O*-galloyl)-hexoside	14.83	711.1216	C_33_H_27_O_18_	3.36	[711]: 559(100)	[711→559]: 389(100)			x	
*Hydroxybenzophenones*											
23	Mangiferin *O*-hexoside	11.84	583.1311	C_25_H_27_O_16_	2.98	[583]: 463(65), 493(100), 565(29)	[583→493]: 331(100)			x	
30	Manguiferin/Isomangiferin	13.85	421.0787	C_19_H_17_O_11_	2.13	[421]: 301(100), 331(94)	[421→301]: 258(100), 273(73)	x	x	x	x
39	Mangiferin/isomanguiferin *O*-gallate (isomer I of II)	15.48	573.0891	C_26_H_21_O_15_	2.82	[573]: 421(100)	[573→421]: 301(100), 331(49)	x		x	
42	Mangiferin/isomanguiferin *O*-gallate (isomer II of II)	15.81	573.0894	C_26_H_21_O_15_	3.36	[573]: 283(44), 403(54), 421(100)	[573→421]: 301(100), 331(56)			x	
46	Manguiferin/Isomangiferin	16.43	421.0794	C_19_H_17_O_11_	6.82	[421]: 301(100), 331(89), 406(58)	[421→301]: 258(100), 273(80)	x		x	
48	Mangiferin-di-*O*-gallate	16.71	725.1004	C_33_H_25_O_19_	2.68	[725]: 573(100)	[757→573]: 403(98), 421(100)			x	
*Flavonoids*											
43	Quercetin-3-*O*-pentosyl-hexoside	15.92	595.1303	C_26_H_27_O_16_	1.49	[595]: 300(100), 301(42)	[595→300]: 255(57), 271(100)			x	
49	Quercetin *O*-hexoside (isomer I of II)	16.82	463.0893	C_21_H_19_O_12_	4.66	[463]: 300(30), 301(100)	[463→301]: 151(67), 179(100)	x		x	x
50	Quercetin *O*-hexoside (isomer II of II)	17.04	463.0898	C_21_H_19_O_12_	5.85	[463]: 301(100)	[463→301]: 151(72), 179(100)			x	
52	Quercetin 3-*O*-pentoside (isomer I of II)	17.54	433.0786	C_20_H_17_O_11_	4.72	[433]: 301(100)	[433→301]: 179(100), 151(80)			x	
53	Quercetin 3-*O*-pentoside (isomer II of II)	17.85	433.0788	C_20_H_17_O_11_	5.22	[433]: 301(100)	[433→301]: 179(100), 151(69)			x	
62	Rhamnetin 3-*O*-pentosyl-hexoside	19.96	609.1467	C_27_H_29_O_16_	1.72	[609]: 299(24), 314(100), 315(49)	[609→314]: 299(100)			x	
68	Rhamnetin 3-*O*-hexoside	21.25	477.1054	C_22_H_21_O_12_	5.51	[477]: 315(100)	[477→315]: 165(100), 193(38), 299(60), 300(44)			x	
71	Rhamnetin	22.05	315.0513	C_16_H_11_O_7_	4.26	[315]: 271(100)	[315→271]: 256(100)			x	

**Table 3:** DPPH and ORAC antioxidant activity.

Sample	DPPH ^1,2^		ORAC ^1,2^
	IC_50_ (μg/mL)	(mmol TE/g Extract)	(mmol TE/g Extract)
*Keitt*			
Skin	11.93 ± 0.69 ^a^	0.47 ± 0.03 ^a^	8.30 ± 0.01 ^a^
Flesh	17.78 ± 0.33 ^b^	0.32 ± 0.01 ^b^	5.20 ± 0.12 ^b^
*T. Atkins*			
Skin	9.97 ± 0.36 ^c^	0.56 ± 0.02 ^c^	11.02 ± 0.11 ^c^
Flesh	22.51 ± 0.44 ^d^	0.25 ± 0.01 ^d^	3.56 ± 0.07 ^d^

^1^ Values are expressed as mean ± S.D. ^2^ Different superscript letters in the same column indicate differences are significant at *p* < 0.05. ORAC: oxygen radical absorbance capacity; DPPH: 2,2-diphenyl-1-picrylhidrazyl method.

**Table 4:** Cytotoxicity of *M. indica* extracts towards gastric (AGS), colon (SW-620), and liver (Hep-G2) carcinoma cells as well as towards Vero non-tumoral cells.

Sample		IC_50_ (µg/mL)			
		AGS ^1,2^	SW 620 ^1,2^	Hep-G2 ^1,2^	Vero ^1,2^
Keitt	Skin	197 ± 16 ^a,b,^*	223 ± 24 ^a,^*^,&^	309 ± 23 ^a,&^	>500 ^a,#^
	Flesh	256 ± 32 ^a,^*	374 ± 18 ^b,&^	369 ± 17 ^a,&^	>500 ^a,#^
T. Atkins	Skin	138 ± 8 ^b,^^	175 ± 7 ^a,^^	164 ± 13 ^b,^^	278 ± 3 ^b,#^
	Flesh	>500 ^c,#^	>500 ^c,#^	>500 ^c,#^	>500 ^a,#^

^1^ Different superscript letters in the same column indicates that differences are significant at *p* < 0.05. ^2^ Different superscript signs in the same row indicates differences are significant at *p* < 0.05.
